# Predation environment affects boldness temperament of neotropical livebearers

**DOI:** 10.1002/ece3.2886

**Published:** 2017-03-26

**Authors:** Josh E. Rasmussen, Mark C. Belk

**Affiliations:** ^1^ Department of BiologyCollege of Life SciencesBrigham Young UniversityProvoUTUSA

**Keywords:** behavioral syndromes, *Brachyrhaphis rhabdophora*, Poeciliidae

## Abstract

Behavioral traits of individuals are important phenotypes that potentially interact with many other traits, an understanding of which may illuminate the evolutionary forces affecting populations and species. Among the five axes of temperament is the propensity to behave boldly in the presence of a perceived risk. To determine the effect of different predatorial regimes on boldness and fearfulness, we assessed the behavior of individuals in a novel portable swim chamber (i.e., forced open‐field test) by *Brachyrhaphis rhabdophora* (*n* = 633). We used an information theoretic framework to compare generalized (logistic) linear fixed‐effects models of predatorial regime (predator‐free [*n* = 6] and predator [*n* = 4] sites), sex, and standard length (SL). Fish from predator sites were much more fearful in the novel arena than fish from nonpredator sites. This varied by length, but not by sex. At 48 mm SL, fish from nonpredator sites were 4.9 times more likely to express bold behavior (ambulation) in the novel swim chamber as fish from predator sites. Probabilities of “ambulating” within the swim chamber increased with size for nonpredator sites and decreased with size for predator sites.

## 1 Background

Directed research of movement behavior as a phenotype is a burgeoning field of research over the past decade (Carter, Feeney, Marshall, Cowlishaw, & Heinsohn, 2013; Dall & Griffith, 2014; Sih, Bell, & Johnson, 2004). Individual behaviors consistent over time or context are referred to as temperament (also called personality by some and used interchangeably here), of which there are generally five recognized axes: (1) boldness–fearfulness; (2) exploration–avoidance; (3) activity; (4) aggressiveness; and (5) sociability (Conrad, Weinersmith, Brodin, Saltz, & Sih, 2011). Temperament is an important component of intraspecific diversity (Magurran, Seghers, Shaw, & Carvalho, 1995; Sutherland, 1996) and has been invoked to explain why animals may exhibit suboptimal behavioral tendencies at times (Carter et al., 2013). Although individuals may alter responses dependent on context, the relative ranking among individuals is generally maintained, that is, some individuals are consistently bolder or more aggressive than others across contexts and time. For example, the population‐level covariance of short‐term temperament measures of sheepshead swordtail (*Xiphophorus birchmanni*; at 4 days after capture) was found to be consistent with measurements taken after 56 days (Boulton, Grimmer, Rosenthal, Walling, & Wilson, 2014).

Given the relative newness of this field of research within animals, there is still regular disagreement on definitions and methods (Beckmann & Biro, 2013; Biro, 2012, 2013; Carter et al., 2013; Dall & Griffith, 2014; Edwards, Winney, Schroeder, & Dugdale, 2013; Réale, Reader, Sol, McDougall, & Dingemanse, 2007). Boldness has been defined as a measure of an individual's reaction to a perceived threat, such as the presence of a predator (Conrad et al., 2011; Réale et al., 2007), or alternatively, as a propensity to take risks, especially in novel situations (Carter et al., 2013). Réale et al. (2007) classify the propensity to explore a novel habitat or object as part of the exploration–avoidance personality axis. While these definitions are similar, the distinction between a novel “situation” and a novel “habitat” can be important and may contribute to a certain level of confusion among studies, as discussed below (Carter, Marshall, Heinsohn, & Cowlishaw, 2012; Misslin & Cigrang, 1986).

To further confound the issue, fear and anxiety are often confused for expressed behaviors. Fear is an emotional reaction to a perceived danger, driven in large degree by the neuroendocrine system (Boissy, 1995), and as such fear itself cannot be measured. Typically researchers quantify the animal's response to the emotion of fear, such as flight distance initiation or plasma corticosterone levels (Stankowich & Blumstein, 2005). In other words, fear is a motivator to which an individual responds with a behavior, dependent on the inherent temperament of the individual (Gray, 1987). The level to which an individual tends to react to fear is characterized across the temperament trait of boldness–fearfulness.

This confounding and confusion of definitions, as well as the inherent complexity of temperament and emotion, present substantial challenges to measuring and interpreting these traits in animals, perhaps especially in noncaptive, nonhabituated individuals (Dall & Griffith, 2014). One of the most common tests utilized is the open‐field test (Boissy, 1995; Carter et al., 2013). This test consists of introducing animals (as individuals or a group, depending on the intent of the research) into an open (i.e., an arena) and novel environment to subsequently quantify the behavior of interest (Brown & Braithwaite, 2004; Burns, 2008; Carter et al., 2013). Measures most typically used include ambulation or distance covered, immobility, and defecation (Burns, 2008; Réale et al., 2007). A couple of variants of this test may actually measure different personality traits: The forced open‐field test places individuals within a novel arena without the option of escape, while the free open‐field test allows for the option of escape into a “refuge” or familiar area, such as a home tank. The former case likely assesses the individual along the boldness axis given the novel situation, while the latter assesses the exploratory temperament of the individual. For example, Misslin and Cigrang (1986) observed that mice did not respond with fear (as measured by blood plasma corticosterone levels) when simultaneously presented novel and familiar environments, but the mice did exhibit signs of anxiety when they were unable to escape the novel environment. The open‐field test was found to be a valid and reliable test of boldness in the related guppy (*Poecilia reticulata*; Réale et al., 2007; Burns, 2008).

We used a forced open‐field test to assess temperament (specifically boldness–fearfulness axis) between predator and nonpredator sites with length and sex as covariates using *Brachyrhaphis rhabdophora*—a tropical member of the livebearing fish family Poeciliidae endemic to continental northwestern Costa Rica (Bussing, 1987). This species provides an excellent subject for this research because it is locally abundant, small‐bodied, and inhabits streams with varying degrees of predation (Johnson & Belk, 2001) providing opportunity for replication within and among the population level easily. We assumed that observed behaviors reflected temperament, recognizing that such behaviors may be motivated by fear due to handling, but that ultimately temperament would in effect translate each individual's fear into quantifiable behavior. We also assumed that the relative order of observed responses would be maintained within and among populations given the consistency of the test across all individuals.

## 2 Methods

**Figure 1:**
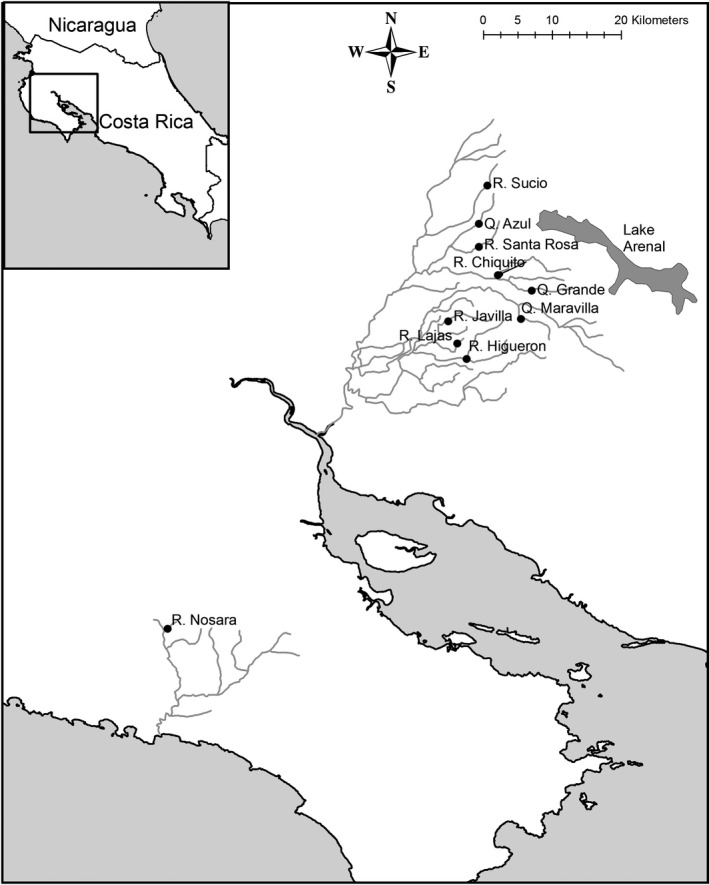
Locality map of study area. Location of ten sites in Costa Rica (four predator and six nonpredator) where fish were sampled for behavioral and morphometric analysis. Rivers Higueron, Javilla, Lajas, and Nosara were classified as predator sites given the presence of the predatorial guapote (*Parachromis dovii*) and the others as nonpredator sites. In the site names, “R.” stands for “Rio” and “Q.” stands for “Quebrada,” terms that loosely translate in English to river and stream, respectively

Adult *B. rhabdophora* were collected during the dry season (January–May) at ten separate locations on the western versant of Costa Rica (Figure 1), with the permission of the Ministerio del Ambiente y Energía, Sistema Nacional de Areas de Conservación of Costa Rica. No voucher specimens were kept as part of this research. Sites were classified as being inhabited by the native piscivorous cichlid guapote (*Parachromis dovii*; called here “predator” environments or sites, *n* = 4) or without guapote (“nonpredator” environments, *n* = 6; Table 1). Native fish communities also included mollies (*Poecilia gillii*), convict cichlids (*Amatitlania nigrofasciatus*), and banded tetra (*Astyanax aeneus*). One site, Rio Chiquito, was also inhabited by the herbivorous characin *Brycon behreae*, which was probably introduced into the area. Competition for food resources between *B. rhabdophora* and the other species may occur; however, as *B. rhabdophora* primarily consumes insects and the others are more typically generalists or herbivorous (the molly and characin, especially), this probably results in weak competitive pressures on the species, if at all.

**Table 1:** Data summary of *Brachyrhaphis rhabdophora* boldness behavior assay, including general site characteristics, and number of bold individuals determined to exhibit fearful behavior in the field behavioral assay (<1 lap) and bold behavior (>1 lap)

Stream	Predator	Elevation (m)	Gradient (m/km)	Sinuosity	Females			Males		
					Fearful	Bold	Total	Fearful	Bold	Total
Quebrada Azul	Absent	485	25	1.10	17	43	60	8	13	21
Quebrada Grande	Absent	364	27	1.32	13	31	44	14	21	35
Quebrada Maravilla	Absent	288	31	1.14	15	11	26	4	16	20
Rio Chiquito	Absent	405	23	1.13	16	17	33	8	10	18
Rio Santa Rosa	Absent	505	29	1.37	8	11	19	1	5	6
Rio Sucio	Absent	378	30	1.41	4	7	11	3	9	12
Rio Higueron	Present	86	19	1.25	24	18	42	8	16	24
Rio Javilla	Present	98	16	1.30	3	9	12	4	5	9
Rio Lajas	Present	73	15	1.14	12	10	22	7	10	17
Rio Nosara	Present	340	1	1.71	22	13	35	13	6	19

The identification of predator type is defined by the presence or absence of the predatorial fish guapote (*Parachromis dovii*).

Reach‐level habitat characteristics differed between predator and nonpredator sites (Table 1). On average (standard deviation), predator sites were at lower elevations than nonpredator sites, 149 (128) m above sea level and 404 (81) m, respectively. Stream gradient and sinuosity were estimated digitally from publicly available aerial photographs by measuring attributes with a 1‐km reach centered on the sampling site. Predator‐free sites were generally steeper than nonpredator sites, dropping on average more than twice as much over one river kilometer: 27.4 (2.9) m/km compared to 12.5 (8.1) m/km for predator sites. Stream sinuosity variability among all sites was relatively uniform (Table 1). Within classifications of the predator dichotomy, these attributes were generally uniform. One exception to this was Rio Nosara, which was at a higher elevation (340 m) with very low gradient (1 m/km) and relatively high sinuosity (1.71), compared to other predator sites.

We collected individuals from ten locations using a handheld seine net approximately 6.0 × 1.5 m with 0.5 cm mesh. The seine net was pulled by two individuals through all potential habitats within the stream until approximately 150 *B. rhabdophora* were captured, between 30 and 60 min for all sites. From this group of fish, all apparently sexually mature individuals were retained. Juveniles were returned to the stream unutilized. Mature males were identified by the presence of a fully developed gonopodium. Females greater than 22 mm (standard length [SL]) were presumed to be sexually mature (Johnson & Belk, 2001).

Males in this study varied between 18 and 42 mm SL ($\overset{¯}{x}$ = 29.2, *SD* = 4.7) and females between 22 and 57 mm SL (($\overset{¯}{x}$ = 34.5, *SD* = 6.5). On average, *B. rhabdophora* from predator sites were approximately 4.1 mm shorter (SL) than their conspecifics from nonpredator sites when adjusted by sex (*F*_1,659_ = 87.7, *p *<* *.0001). This effect was stronger for females (4.9 mm difference on average; 95% confidence interval 1.5–8.2 mm) than for males (2.7 mm difference; 1.2–4.2 mm). The ratio of males to females was similar across predator and nonpredator sites (χ^2^ < 0.01, *p *≈* *1). However, females outnumbered males overall (442–221, respectively).

The propensity of adults (*n* = 663) to behave boldly (ambulation) in the novel arena was assayed in a portable swim chamber constructed from a white five‐gallon bucket. Upon capture, fish were immediately transferred to an opaque holding tank until they were removed individually for assay, between 30 and 180 min after capture. A smaller, weighted bucket was placed in the center to create a circular swim track approximately 100 mm in depth with a minimum (i.e., central) circumference of 440 mm and a maximum (i.e., peripheral) circumference of 839 mm (see Rasmussen & Belk, 2012 Figure 2 for a detailed description of swim chamber). Inconspicuous marks were placed around the periphery of the swim chamber to facilitate estimation of distance moved by subjects during the test. Subjects were placed inside the swim chamber and allowed to acclimate for 2 min. We presumed that extreme fear responses were accounted for by this brief acclimation period based on a priori tests to determine when initial frantic escape behaviors typically ceased. After the acclimation period, we observed whether each individual travelled at least one lap around the swim chamber, based on a priori tests suggesting a dichotomy here. Individuals completing <1 complete lap were classified as a “fearful” (*n* = 283, 42.7%), and those completing ≥1 lap were classified as “bold” (*n* = 380, 57.3%).

**Figure 2:**
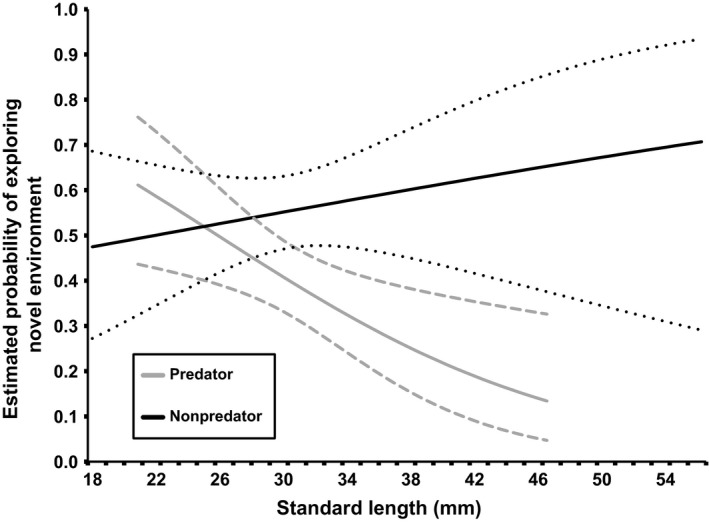
The probability of exhibiting boldness within a novel environment. The probability of exhibiting boldness within a novel environment (portable swim chamber) as a function of length, based on the most supported model. Dashed lines represent 95% confidence intervals. Values presented here are for females; however, as no significant interaction effects including sex were found, these values are generally representative of males as well. The order term was fixed at the median value of 34

After completion of the behavioral assay, individuals were allowed to recover in fresh water prior to being returned to the stream. All animals were handled in accordance with programmatic ethical techniques approved by the Institutional Animal Care and Use Committee of Brigham Young University (Provo, UT).

### 2.1 Statistical analysis

The dependent variable was modeled as a binomial variable designating whether each individual fish was classified as bold (1) or fearful (0). Based on a priori consideration of hypotheses of factors that may affect this behavior, several independent variables and interactions were identified, including two categorical factors: predatorial regime (predator environment = 1) and sex (female = 1). Plausible biological reasons could be given for considering two aspects of individual length: absolute SL and relative length (a standardized metric to capture size relativity by calculating the *z*‐score for each individual within sex at each site). This relative length metric permits a standardized assessment of individual length relative to other individuals captured at the same site, which can be affected by predation and other factors (Johnson & Belk, 2001). However, inclusion of both factors in a single model would certainly cause multicollinearity (*R* ^2^ = .64 and variable inflation factor >6.5 for both absolute and relative size), causing erratic coefficient estimation and standard error inflation. Therefore, no models included in the analysis contained both of these factors simultaneously.

Additionally, a Mann–Kendall trend test suggested that the order in which individuals were assayed may not have been entirely random. Five of the ten sites (four nonpredator and one predator) produced significant (<.05) *p*‐values, indicating a bias toward larger individuals being tested earlier despite attempts for random ordering. This could result in bias as smaller fish tend to have higher metabolisms which may prompt them to seek food sooner than larger individuals, even under stressful conditions (Brown & Braithwaite, 2004). Similar lack of randomness was detected between males and females with a two‐sided Wilcoxon rank sum test. At three nonpredator sites and one predator site, females were generally tested earlier than males, which corroborates the Mann–Kendall test, given that females tend to be larger than males. To statistically control for potential variation introduced by these patterns (e.g., longer time in the holding tank for males than females or smaller individuals generally), an ordinal parameter (Order) was included in the models to statistically control for better estimation of the parameters of interest, but was essentially a nuisance variable. The rank for each individual was determined simply as their order of testing within each site. This parameter was treated as a continuous covariate for each individual.

Several interactions among these parameters were also presumed to have biological support, including predator regime by length (either SL or *z*‐score) or sex, sex by length, order by length, and predator regime by sex and length. No biologically plausible hypotheses were presumed to support order by predator regime or order by sex, so these interactions were not included in candidate models.

To assess differences in the probability of the bold phenotype being expressed, we developed a set of candidate generalized linear models with a logit link function (*n* = 85). Maximum likelihood estimation of coefficients and variance components was performed using the *glmer* function from the *lme4* package (Bates, Maechler, Bolker, & Walker, 2015) within R (R Core Team 2014). We utilized an Information Theoretic approach (i.e., Akaike's information criterion [AIC]; Akaike, 1973; Burnham & Anderson, 1998) to compare the relative strength of each candidate model given the data. The global model, that is, the most parameterized, included all main effects and all possible interaction terms, including a three‐way interaction. Overall estimates of goodness of fit of this global model suggest that fit was adequate (Hosmer–Lemeshow $\hat{C}=9.60$, *p *=* *.38, *df* = 9). The null hypothesis of this test is that the model is an adequately correct model. Therefore, rejection of the null hypothesis suggests that model fit is unacceptable. This statistic is based on the comparison of observed values to expected values of groups of individuals (e.g., deciles) with similar expected values (i.e., fitted probabilities) and is compared to a Chi‐squared distribution with degrees of freedom one less than the number of groups. Overdispersion of the data was also minimal ($\hat{c}$ = 1.07); therefore, AIC and variance values were not adjusted to account for overdispersion in any way (Burnham & Anderson, 1998; Hosmer & Lemeshow, 2000).

## 3 Results

Given our model set and data, only 33 models of the 85 were determined to have substantial support in the data (i.e., ΔAIC ≤ 2.0). Furthermore, the ΔAIC values within the “supported” set suggest that there was relatively strong support for the “best” model (Table 2): Predator × SL + Sex + Order × SL (including the main effects of the interaction terms). This most supported model accounted for nearly 30% of the model weights (when compared to the entire model set) and had roughly 2.6 times support from the data than the two other “supported” models, which only differed from the most supported model by the addition an interaction between sex and SL or between predator and sex, respectively. These three models accounted for over half of the model weight within the original model set (Table 2). All other models ranged from approximately 3.0 times to >1,000 times less likely than the most supported model. Additionally, the area under the receiver operator curve suggests discrimination by these three models is relatively good. This metric suggests that any of these supported models would make accurate predictions roughly 83% of the time on a new set of data.

**Table 2:** The top 3 supported model set and information theoretic values for each model given the a priori set and data

Model description	Number of parameters	AIC	ΔAIC	Model likelihood	Model weight	Cumulative weights	Evidence ratio
Predator × SL + Sex + Order × SL	7	674.34	0.00	1.00	0.295	0.295	1.0
Predator × SL + Sex × SL + Order × SL	8	676.25	1.91	0.39	0.114	0.408	2.6
Predator × SL + Predator × Sex + Order × SL	8	676.34	2.00	0.37	0.108	0.516	2.7

SL is standard length. The predator factor refers to the presence or absence of the predatorial cichlid guapote (*Parachromis dovii*). Models with ΔAIC > 2.0 were excluded from further consideration because of relatively little support from the data. Cumulative weights were calculated across the entire set of candidate models (*n* = 85).

The standardized metric of relative size (*z*‐score) was not included in any well‐supported model, but all other main effects were in every well‐supported model, as were interaction terms between predator and SL and between order and SL, giving each of these terms a relative importance of 1.0 (Table 3). The other two interactions considered (predator by sex and sex by SL) had relatively little importance in the well‐supported set (a relative importance of roughly 0.21). The order term affected the deviance of the best model roughly 10 times more than the next most influential parameter (predator regime; Table 3). Sex was the weakest main effect.

**Table 3:** Model‐averaged values of each parameter included in the analysis set of models

	Intercept	Predator	Sex	SL	Order	Predator/SL	Predator/sex	Sex/SL	Order/SL
Model‐averaged value	−0.83	2.90	0.48	−0.031	0.01	−0.12	0.01	0.48	0.002
Parameter relative importance	1.00	1.00	1.00	1.00	1.00	1.00	0.21	0.22	1.00
Model‐averaged variance	1.58	1.42	0.25	0.001	0.001	0.002	0.20	0.002	<0.001
Adjusted standard error	1.26	1.19	0.50	0.04	0.03	0.04	0.45	0.04	0.001
Lower 95% interval	−3.30	0.57	−0.49	−0.11	−0.05	−0.19	−0.87	−0.10	0.000
Upper 95% interval	1.64	5.23	1.46	0.04	0.06	−0.04	0.89	0.07	0.003
Drop in deviance	NA	19.8	3.2	12.8	219.9	9.6	NA	NA	4.10

Values were calculated based on standard model‐averaging methods (Burnham & Anderson, 1998). Only parameters found in the analysis set (i.e., ΔAIC ≤ 2.0) are presented here because all other parameters had little support in the data: predator (predator sites = 1); sex (female = 1); SL = standard length; order is an ordinal factor (1:*n* for each site) describing the order in which each individual was assayed at a given site. The drop in deviance value is the difference between the deviance of the most supported model and the deviance of a model that lacks just that parameter. Drop in deviance for main effects includes the exclusion of any related interaction terms.

In general, individuals from nonpredator sites were more likely to be classified as bold compared to predator sites (Figure 2); however, predictions overlapped broadly at smaller sizes. The probability of behaving boldly declined steadily for predator sites as length increased, but steadily increased with size at nonpredator sites (Figure 2). At the largest overlapping sizes (46.9 mm SL), an individual from a nonpredator site is 4.9 (95% confidence interval 2.6–8.0) times more likely to behave boldly within the novel swim arena as an individual of the same size from a predator site, as predicted by the best supported model. The other two models in the “well‐supported” set suggest that females tended to have higher probabilities of boldness within the swim chamber than males when compared within predator site classification, but confidence intervals between the sexes broadly overlap across all sizes.

## 4 Discussion

Phenotypic diversity is an important component of the ecology of any species, and behavioral phenotypes (commonly referred to as temperament or personality) are part of the suite of individual characteristics that can be considered to better understand population‐level dynamics (Sih et al., 2004). Diversity in characteristics typically arises from a complex interplay of extrinsic and intrinsic factors, that is, environmental conditions and genotype, often with trade‐offs involved (Cote, Fogarty, Tymen, Sih, & Brodin, 2013; Sih et al., 2004). One of the common axes of temperament is the boldness–fearfulness axis, which is defined as the reaction of an individual to a perceived threat or danger (Conrad et al., 2011). Diversity within this personality trait has the potential to influence other broadly important processes such as dispersal (Baguette, Stevens, & Clobert, 2014; Rasmussen & Belk, 2012; Thorlacius, Hellstrom, & Brodin, 2015), fitness (Réale et al., 2007), and adaptation (Sih, Cote, Evans, Fogarty, & Pruitt, 2012). Consequences of fearfulness may include less opportunity to optimize habitat, food, or mates leading to divergent feeding and possibly mating behaviors (Gilliam & Fraser, 1987; Sih, 1997; Skalski & Gilliam, 2002). In summary, our results demonstrate that the propensity of *B. rhabdophora* to behave boldly given the threatening situation of an unescapable novel environment is affected by both extrinsic (predator regime) and intrinsic (length) factors.

Biro (2012) asserted that within‐individual responses to behavioral tests are not consistent in the first 2 days after individuals have been stressed and cautioned that the rapid assay of individuals in a field environment may misclassify individuals. These conclusions were based on repeated measurements Ward's damselfish (*Pomacentrus wardi*) that only became consistent after the first 2 days within captivity. These assertions are compelling, but are not entirely applicable to boldness for a couple of reasons. First, given that boldness characterizes the expressed behavior when threats or dangers are perceived, therefore, stress, fear, or anxiety must be part of the test of boldness. These can be induced by presenting the subject with a known threat such as a known predator or by introducing the subject into a novel environment, as was done here. Secondly, repeated behavioral assays may habituate individuals to handling or a situation, which may be somewhat contrary to the intent of assessing some behaviors, particularly boldness given a perceived threat. As described by Edwards et al. (2013), repeated assays in an increasingly familiar environment conflate the distinction between the boldness and exploratory personality traits. Biro (2013) subsequently acknowledged that valid challenges could be made to his previous position (Biro, 2012) given the prevalent differences in how to design valid experiments to assay temperament.

Beckmann and Biro (2013) also questioned the reliability of any single assay of behavior in temperament research based on their finding of a lack of correlation of a single emergence test with individuals from two damselfish species (*P. wardi* and *Pomacentrus ambionensis*) compared to two other tests conducted in the individual's home tank. A single behavioral assay requires an assumption that the observed behavior will be consistent with behavior within the same behavioral axis in other contexts (Carter et al., 2013; Réale et al., 2007); otherwise, it cannot be considered temperament (Beckmann & Biro, 2013). This emphasizes the concept that, though personality is consistent across time and context, consistency is best captured not in the “absolute value” of the measure, but in the relative rank order of the test subjects, given that the “absolute value” of a behavior can vary dependent on the magnitude or type of the stimuli. Given this, we contend that our single assay of boldness is a valid and reliable measure of the relative prevalence of boldness among populations with different predatorial regimes.

Fish from predator sites were much less likely to be classified as bold, at least for average to larger‐sized individuals, but we cannot specifically separate the effects of the sympatric predator from the suite of confounding habitat differences between predator and nonpredator sites, but the practice of identifying predator and nonpredator environments to draw ecological and evolutionary conclusions is a common practice with this species and similar species (Fraser, Gilliam, Daley, Le, & Skalski, 2001; Ingley, Billman, Belk, & Johnson, 2014; Jennions & Telford, 2002; Johnson & Belk, 2001; Langerhans, Layman, Shokrollahi, & DeWitt, 2004; Reznick & Endler, 1982). Inclusion of more detail regarding the site (e.g., continuous stream temperature or discharge measurements) would not be relevant in this instance. It is entirely probable that these factors could influence the temperament of *B. rhabdophora* over the long term (lifetimes to generations); nevertheless, single‐point measurements or even limited continuous sampling would likely be uninformative for a couple of reasons: (1) Limited environmental stream data would not potentially capture the relevant variation that produces effects from environmental conditions on temperament over the long term and (2) given the study design, stream point data would be information poor because all of the individuals at a given site would be associated with the same measurement value as every other individual from that site.

Predation effects were the strongest predictor of expression of fearfulness in novel situations of *B. rhabdophora*. This is likely a direct result of the removal of less fearful individuals from areas where predation is higher due to the presence of the predatorial cichlid. However, the question whether this difference in temperament is heritable remains. Certainly, evolutionary theory predicts that direct selective pressure will ultimately select against boldness within the “predator” populations, suppressing the prevalence of the behavior compared to some baseline. Genetically based life history differences in size and age of maturity due to predation in this species and closely related species (e.g., *P. reticulata* and *Gambusia affinis*) are well documented (Ingley, Billman, et al., 2014; Johnson & Belk, 2001; Langerhans et al., 2004; Reznick & Endler, 1982). Specifically, Johnson (2001) showed that these predation‐induced differences in *B. rhabdophora* had a genetic basis. Dingemanse et al. (2009) concluded that both individual experience (i.e., information) and evolutionary history (i.e., selection) of predation affected expression of personality traits of three‐spined stickleback (*Gasterosteus aculeatus*) by direct selection and by influencing the expression of heritable variation. However, current animal personality theory predicts that individual temperament would be consistent over time and context. If the phenotype were entirely genetically based, we may expect that the differences between predator and nonpredator populations would be more or less uniform across the spectrum of lengths rather than just at the longer lengths. Possible alternatives include social learning (observing a predation event for example; Brown & Laland, 2002; Johnson & Basolo, 2003). In the case of Johnson and Basolo (2003), female green swordtails (*Xiphophorus helleri*) preferred males with digitally shortened swords over the typical preference (brightly colored, long swords) if they had witnessed a male with a long sword being predated upon. Our study design does not make it possible to draw conclusions about how likely each of these alternatives is.

In general, overall length was an important factor in determining the probability of exhibiting bold behavior, although the patterns were different depending on predator classification. Larger individuals from predator sites were less likely to be classified as bold than smaller fish. Similarly, Brown and Braithwaite (2004) found rates of boldness, for example, time until exiting refuge habitat given the presence of a potential threat, of *Brachyrhaphis episcopi* to be size based, a phenomenon they attributed to relatively higher metabolic requirements of smaller fish which tended to emerge from cover sooner than larger individuals. They also found, however, that predation risk tended to decrease boldness of smaller fish compared to nonpredator sites. Similarly, *Brachyrhaphis roseni* and *Brachyrhaphis terrabensis* were found to be less bold when from populations that co‐occur with predators, but sex, not size, was found to affect boldness (Ingley, Rehm, & Johnson, 2014; Money, Ingley, & Johnson, 2017). The metabolic hypothesis may explain why smaller fish from predatorial sites appear to be less fearful than larger fish from the same sites in our study, especially since smaller individuals should display more fearful behavior than larger individuals because they are generally more susceptible to predation (Brown & Braithwaite, 2004). However, it does not explain why larger individuals from nonpredator sites should exhibit bolder behavior.

## Conflict of Interest

None declared.
